# Remodeling the ECM: Implications for Metastasis and Tumor Dormancy

**DOI:** 10.3390/cancers13194916

**Published:** 2021-09-30

**Authors:** Julie S. Di Martino, Tasmiah Akhter, Jose Javier Bravo-Cordero

**Affiliations:** The Tisch Cancer Institute, Icahn School of Medicine at Mount Sinai, New York, NY 10029, USA; julie.dimartino@mssm.edu (J.S.D.M.); ztakhter-20@hsmse.org (T.A.)

**Keywords:** ECM, cancer dormancy, metastasis

## Abstract

**Simple Summary:**

In this perspective article we discuss the importance of investigating the role of the ECM on tumor metastasis and tumor dormancy. First, we discuss the importance of the ECM in cancer progression. Then, we revise the parallelisms between dormant cells and stem cells and the role of the ECM in stem cell regulation. Finally, we comment on the evidence describing the role of the ECM in tumor dormancy and highlight key outstanding questions in the tumor dormancy field.

**Abstract:**

While most primary tumors can be effectively treated, therapeutics fail to efficiently eliminate metastases. Metastases arise from cancer cells that leave the primary tumor and seed distant sites. Recent studies have shown that cancer cells disseminate early during tumor progression and can remain dormant for years before they resume growth. In these metastatic organs, cancer cells reside in microenvironments where they interact with other cells, but also with the extracellular matrix (ECM). The ECM was long considered to be an inert, non-cellular component of tissues, providing their architecture. However, in recent years, a growing body of evidence has shown that the ECM is a key driver of cancer progression, and it can exert effects on tumor cells, regulating their metastatic fate. ECM remodeling and degradation is required for the early steps of the metastatic cascade: invasion, tumor intravasation, and extravasation. Similarly, ECM molecules have been shown to be important for metastatic outgrowth. However, the role of ECM molecules on tumor dormancy and their contribution to the dormancy-supportive niches is not well understood. In this perspective article, we will summarize the current knowledge of ECM and its role in tumor metastasis and dormancy. We will discuss how a better understanding of the individual components of the ECM niche and their roles mediating the dormant state of disseminated tumor cells (DTCs) will advance the development of new therapies to target dormant cells and prevent metastasis outgrowth.

## 1. Introduction

Metastasis is a deadly disease. Despite the tremendous progress that has been made treating primary tumors, prevention of metastasis is limited. Metastasis results when disseminated tumor cells (DTCs) awaken from a dormant state where they often remain for decades [1]. Dormancy is often noted as one of the most threatening aspects of cancer, as it is asymptomatic and undetectable, but can eventually lead to the development of aggressive metastases. Studying the interaction of dormant DTCs with their microenvironment and particularly the extracellular matrix (ECM) is challenging but will improve our understanding of how dormancy niches are regulated. This knowledge will pave the path for the development of new therapies to eliminate dormant cells by targeting their ECM microenvironment. A strategy that could ultimately lead to permanent control over cancer relapse and hope for a treatment to prevent metastasis.

Cancer dormancy is a relatively new field and knowledge of the biology of dormant cells is limited. Dormant cells are characterized by (i) a deep state of quiescence, in which growth is reversibly arrested at the G_0_/G_1_ cell cycle phase, (ii) an induction of dormancy-associated genes (*BHLHE41*, *NR2F1*, *CDKN1B*, *CDKN1A*); (iii) activation of p38 signaling and inhibition of proliferative ERK signaling; (iv) increased survival via activation of the unfolded protein response (UPR) pathway and expression of survival genes (*HSPA5*, *DDIT3*); (v) latent pluripotency via *NR2F1* upregulation; and (vi) epigenetic reprogramming [2,3,4]. Thus, cancer dormancy is defined as a molecular program involving deep quiescence, survival and latent pluripotency [5,6]. These features are shared by dormant stem cells [7,8] but not slow-cycling cells. Importantly, dormant cells differ from senescent cells as their G_0_ state is reversible.

The dormant cell state allows cancer cells to escape chemotherapy [9] and survive for decades at sites distant from the primary tumor. These sites have been defined as dormancy niches and have been characterized in bone marrow [10,11,12,13] as well as other organs (i.e., lungs [14]).

Dormant cells lie in silence and are restored to growth when conditions become optimal. Interestingly, it has been reported that some recipients of organ transplantation have developed their donor’s tumors [15,16,17]. The donors, who had no tumors, carried dormant and undetected cancer cells that were awakened in the recipient patient. We can hypothesize that the recipient patient microenvironment (modified by immunosuppressive treatments) facilitated the transition. These results highlight that the microenvironment plays a role in the maintenance as well as the escape of DTCs from their dormant state.

The tissue microenvironment is composed of a cellular component (including immune cells, fibroblasts, adipocytes) and an acellular component: the extracellular matrix. For a very long time, the ECM was described as the gap-filling matter that helped to support different shapes and levels of stiffness to different organs of the body. While this is true, the ECM plays a far greater variety of roles, and it is, in fact, a very plastic and dynamic compartment that can direct ligands to surface receptors in cells and also act as a storage site that permits regulated and controlled release of growth factors, chemokines and cytokines that can modulate cellular behaviors (growth, migration) and ECM remodeling. ECM molecules can signal to stromal and cancer cells to promote or restrict cancer progression. For example, increased deposition of collagen I increases the risk of breast cancer by 4 to 6 fold [18]. On the other hand, encapsulated hepatocellular carcinoma, in which the capsule is enriched with collagen I, is a better prognostic indicator in patient survival [19,20], while mutations in the ECM can lead to severe diseases such as osteogenesis imperfecta (mutation of COL1A1, COL1A2), Ehlers-Danlos syndrome type IV (mutation of COL3A1), and epidermolysis bullosa (mutation of COL7A1), just to cite a few [21].

Proteomics-based studies of the ECM proteome conducted by Dr. Richard Hynes’ laboratory unveiled an unexpected variety and diversity of proteins constituting the ECM of tumors and metastasis [22,23] (http://matrisomedb.pepchem.org/ accessed on 10 September 2021). The resulting signature (named the matrisome) constitutes more than a thousand proteins and varies by tissue (visit matrisome.org for an exhaustive list of matrisome signatures). It is defined as the ensemble of ECM and ECM associated proteins, and is composed of a core matrisome (glycoproteins, collagens, and proteoglycans) and an associated matrisome (ECM regulators, ECM related proteins, and secreted factors).

The ECM is a complex multi-functional compartment and can influence multiple biochemical processes; it functions as an adhesive substrate, provides essential structure, controls inter-cellular communication, and presents growth factors to their receptors regulating cell processes such as proliferation, migration, invasion, and differentiation. ECM molecules can assemble in different types of meshwork to provide different textures and functions to tissue. For instance, the ECM can organize as an impermeable basement membrane separating epithelial cells (or endothelial cells in the case of blood vessels) from connective tissue (or interstitial matrices) where cells (fibroblast, immune cells) reside intercalated within fibrous ECM. In tumors, cancer cells can break through the basement membrane and invade connective tissue, which can then act as pro- or anti-tumoral tissue depending on its composition and organization.

In this perspective we will present the supporting literature to emphasize that a greater degree of urgency should be given to the investigation of the roles played by ECM in cancer dormancy. We will first give an overview of what is known, then we will comment on recent work that shows how the ECM is implicated in the regulation of dormancy and the parallel role of the ECM on stem cell quiescence. Finally, we will discuss the importance of increasing research in this specific field.

## 2. ECM and Cancer Progression

A large body of evidence has shown that the ECM can influence the hallmarks of cancer defined by Hanahan and Weinberg twenty years ago [24,25]. From promoting tumor growth, to resisting apoptosis, to stimulating angiogenesis and priming cancer cell migration and invasion, the ECM can affect a variety of cellular processes that will promote cancer progression.

Early work from Dr. Mina Bissell’s laboratory demonstrated that inhibition of breast cancer cell adhesion to the ECM using a beta-1 integrin inhibitor could reverse a malignant phenotype and restore cancer cells to a normal phenotype, capable of assembling a typical basement membrane [26]. This study demonstrates that adhesion to the ECM can drive cancer cells toward a more aggressive behavior.

In addition to composition, ECM architecture also plays a major role in determining cancer outcomes [27]. Pioneering work from Dr. Patricia Keely’s laboratory identified a tumor-associated collagen signature-3 (TACS-3) that is prognostic of a poor outcome. Such collagen is aligned and this architectural signature, which can be clearly distinguished from the wavy ECM observed in normal tissue and benign tumors, can be used to identify invasive tumors. It has been shown that cancer cells can use these aligned fibers as cables to migrate away from the primary tumor [28] as well as to promote intravasation [29], the step in the metastasis cascade that is key for cancer cells to disseminate.

ECM crosslinking has also been linked to tumor progression [30]. Lysyl oxidase enzymes serve to crosslink collagens and elastin, which increases tissue stiffness. Crosslinked collagen cannot be easily degraded and as a result, collagen accumulates and produces tissue fibrosis, a perfect environment for initiating and promoting cancer development. For example, liver fibrosis increases the risk of developing hepatocellular carcinoma [31].

Individual ECM components such as collagen I can also stimulate cancer cells in a cycle that enhances ECM degradation by matrix metalloproteases (MMPs) and promotes local tumor invasion [32]. The actions of MMPs are generally associated with cancer progression. For instance, MMP10 expressed in stromal cells can promote the invasion of cervical and bladder cancer cells [33]. While MMP9 produced by cancer cells drives the development of metastasis in triple negative breast cancer [34] and can promote circulating tumor cells to colonize the lungs in luminal B breast cancer [35].

**Figure 1:**
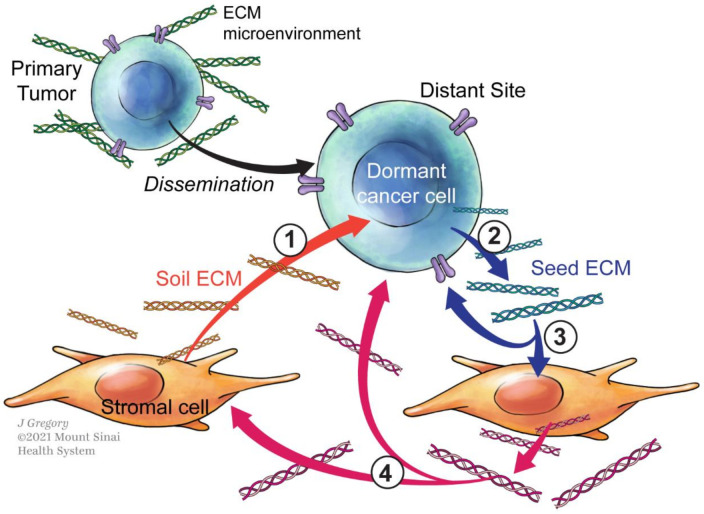
The seed at the center of Paget’s theory. Cancer cells (represented in blue) disseminate to a distant site. (**1**) The seed (cancer cells) will first sense and attach to the “Soil ECM” (represented in yellow). (**2**) The seed will also secrete the “Seed ECM” (blue). (**3**) In the context of dormancy, this “seed ECM” will sustain the dormant state of the seed itself and influence the stromal cells to adapt their ECM production (red). (**4**) This ECM produced by stromal cell (red) in response to the dormant cells will then be able to signal back to the stroma itself and to the seed.

Cancer cells can also produce their own ECM, contributing directly to the shape and content of ECM in the tumor microenvironment [22]. While ECM is mainly produced by fibroblasts in normal and pathological conditions, it is important to highlight that cancer cells (and other cell types) are also capable of generating and secreting matrix [36]. Such tumor-derived ECM can act in an autonomous and non-autonomous manner, influencing the surrounding stromal cells, and changing the surrounding ECM composition and structure in a way that ultimately impacts stromal and tumor cell responses. On the other hand, stromal cell-secreted ECM may affect cancer cells in a non-cell autonomous manner regulating their cellular behaviors (growth, invasion) (Figure 1).

Several studies have highlighted the importance of tumor-derived ECM on cancer cell behaviors. For example, P-cadherin induces secretion of Decorin, a proteoglycan that is essential for collagen fibrils assembly. The core of Decorin binds to type I collagen fibrils and extends its glycosaminoglycan chain laterally toward adjacent collagen fibrils in order to create an interfibrillar structure that promotes collagen fibrillogenesis and fiber alignment, and supporting directional collective migration of cancer cells toward nearby blood vessels [37]. Highly metastatic breast cancer cells increase their production of hyaluronic acid [38], which accumulates at the surface of metastatic cancer cells and binds to CD44 at the plasma membrane [39]. During cancer cell invasion, the repertoire of laminin proteins at the plasma membrane changes. Cancer cells at the leading edge of invasion express a component of laminin 5, the gamma 2 chain gene [40], which helps them to anchor to the ECM during migration and invasion of the normal stroma [41]. Another ECM protein, SNED1 is secreted by cancer cells to promote metastasis in breast tumors [36]. Overall, these studies suggest a coordinated mechanism between ECM secreted by cancer cells and the ECM binding receptors to adapt their cellular behaviors.

These studies show that ECM composition, architecture, crosslinking, accumulation, and degradation seem to be crucial for cancer promotion and progression.

## 3. ECM and Stem Cells Quiescence

Stem cells are pluripotent cells, which under given conditions will divide to produce daughter cells. Daughter cells go on to become stem cells themselves as a part of self-renewal mechanisms or they progress to a more differentiated state in order to replace cells or generally contribute to an organism’s homeostasis. Adults stem cells remain mostly quiescent for periods extending to lifetimes occupying stem cell niches such as bone marrow (hematopoietic stem cell niche), skin, hair follicles, or intestinal crypts [42].

The ECM is an important component defining a stem cell niche, and there are strong parallels between tumor cell dormancy and adult stem cell quiescence [13]. Recent literature has shown that the ECM is essential for regulating stem cell maintenance, proliferation, and self-renewal [43]. In the context of cancer, studies have shown that DTCs reside in a similar bone marrow perivascular niche to stem cells and can be mobilized by agents similar to the ones used to mobilize hematopoietic stem cells [44,45]. Several studies support the notion that ECM molecules play major roles in the stem cell niche construction and the maintenance of their quiescence phenotype such as collagen IV and collagen VI, for adult hematopoietic stem cells or collagen V for quiescent muscle stem cells [43,46,47].

It has been described recently that collagen V is critical for maintaining the quiescence of muscle stem cells. Adult muscle satellite cells produce collagens to maintain quiescence in a cell-autonomous manner using the Notch pathway. Downregulation of the Notch pathway due to a lack of collagen V, favors stem cell exit from their quiescent state [48]. In the bone marrow, Periostin, a secreted ECM protein, promotes maintenance of quiescent hematopoietic stem cells via integrin signaling in an outside-in mechanism [49]. The interaction between Periostin and Itgαv inhibits the FAK/PI3K/AKT pathway, resulting in an increase in p27 expression, which is necessary to inhibit the cell cycle and to maintain quiescence. In hair follicles, stem cells are maintained in a quiescent state by inverse laminin (LN)-332 and LN-511 gradients within the basement membrane. Deposition of LN-332 and LN-511 is mediated by integrin linked kinase [50]. LN-332 suppresses Wnt signaling, whereas LN-511 promotes TGF-β signaling.

ECM molecules also play a role in stem cell self-renewal. For instance, COL VI regulates muscle satellite cell self-renewal [46] and VCAM-1 regulates self-renewal of HSC [51].

Importantly, these ECM components, relevant in the stem cell niche context, are also enriched in ECM from dormant cancer cell origins (Di Martino et al., unpublished data), suggesting that cancer cells may construct a stem cell-like ECM niche to sustain their dormant phenotype. These ECM components seem to trigger similar signaling pathways in dormant cancer cells such as activation of p27, Wnt and TGFβ pathways as observed in stem cells [2,52,53,54]. Recent work by Dr. Aguirre-Ghiso’s laboratory shows that the hematopoietic stem cell (HSC) dormancy niches regulate breast cancer dormancy. NG2+/Nestin+ mesenchymal stem cells maintain the dormancy of both HSC and DTC in the bone marrow through TGFβ2 and BMP7 [55]. These results highlight the parallels between stem cell quiescence and tumor dormancy.

## 4. Role of ECM on Tumor Dormancy: What Do We Know?

The literature in the field of ECM and dormancy is very scarce. Nevertheless, there are some studies that suggest that the ECM might play a role in determining DTC fate: either by sustaining dormancy or favoring the awakening and metastatic outgrowth. As we discussed earlier, changes in ECM can lead to cancer progression, suggesting there may be pro-dormancy ECM molecules. We can hypothesize that, similar to what it is described in the metastatic niche, where proteins such as tenascin C [56] favor metastatic growth, the enrichment of a specific ECM molecule in the microenvironment could lead to DTC dormancy.

On the other hand, as ECM homeostasis is highly regulated by cycles of ECM synthesis, remodeling and degradation [57], a dysregulation in this cycle by increasing production of ECM, or failure to degrade it, would also be anticipated to perturb ECM microenvironment homeostasis and potentially affect DTC behavior.

Several studies have shown that ECM molecules contribute to dormancy and reactivation. Pioneering work by Dr. Liliana Ossowiski’s laboratory showed that dormant cells lose integrin activation and do not assemble fibronectin fibrils in vivo in head and neck squamous cell carcinoma dormancy models, contributing to ERK inhibition and p38 activation [58]. Recently, it has been shown that dormant cells can organize fibronectin to maintain dormancy in breast cancer cell lines [59]. Similarly, in ER+ breast cancer cells, fibronectin increases the number of dormant cells in the presence of FGF-2 [60,61] in the context of bone marrow dormancy acting as a pro-survival signal. These results suggest that an ECM molecule may play multiple roles in the context of dormancy depending on the localization and the tumor type studied.

On the contrary, integrin activation via interactions with collagen I mediates an escape from dormancy [14]. Periostin [62,63,64] and tenascin C [65,66,67] promote metastasis whereas osteopontin supports leukemia cell dormancy [68]. Treatment of mice with an osteopontin-neutralizing antibody to target leukemia cell–ECM niche interactions sensitize dormant cells to chemotherapy highlighting the potential of targeting ECM-cancer cell interactions to kill cancer cells.

Previous work in breast tumors has shown that enrichment in type I collagen allows the awakening of the dormant cell line D2.OR (murine breast cancer) in 3D-cultures grown in vitro and in mice, lung fibrosis (accumulation of collagen) significantly increases total tumor burden in the mice lungs [14]. These strong correlations support a direct role for type I collagen in dormant cell awakening. Moreover, the expression of an ECM crosslinking gene, LOXL2, in DTCs induces epithelial to mesenchymal transition (EMT) and promotes transition from dormancy to proliferation in a basement membrane enriched in vitro system, by acquisition of a stem-like phenotype [69]. In this study, the authors also show in vitro that cells that were able to retain an epithelial phenotype remain dormant. These data associated with the fact that EMT has recently been associated with an epigenetic signature of ECM remodeling genes [70] demonstrate that ECM remodeling and degradation play a role in determining DTC fate.

Recent work by Aguirre-Ghiso’s laboratory has shown [71] that DTCs retain mesenchymal traits at early stages of the dissemination process while they seed as dormant cells in distant sites. Dormant disseminated cancer cells are E-Cad^low^ while overt metastases are E-Cad^med-high^, a level that serves to restore their epithelial identity [54]. Interestingly, work from Dr. Ewald’s laboratory recently showed that E-Cadherin expression is required for metastasis [72]. Based on these data, we hypothesize that cancer cells may need to acquire mesenchymal traits in order to escape the primary tumor. They retain these mesenchymal traits at early stages of the dissemination process. Over time, disseminated dormant cancer cells will regain epithelial traits allowing them to escape dormancy and grow metastases.

There is also growing evidence of the role of MMPs in dormancy. It was recently shown that cancer cells entering dormancy use integrin receptors to secrete and organize a fibronectin meshwork via MMP2-mediated fibronectin degradation [59]. In addition, recent work by Egeblad’s laboratory [73] showed that digestion of laminin-111 via MMP9 by neutrophils contributes to the awakening of dormant breast cancer cells.

On the opposite side, some ECM components have been described as pro-dormancy. Matrigel is a cell- and tissue-derived ECM commonly used to cultivate cells in vitro. It has been shown to be able to slow down the growth of lung cancer A549 cells in vitro [74]. Aligned with the same concept, basement membrane rich substrates can decrease proliferation of HT1080 cells, suggesting an ECM-induced dormancy [75]. Furthermore, ECM binding to syndecan has been recently identified as responsible for growth arrest of DTCs in distant organs [76].

Altogether, this evidence suggests that DTCs are not passive seeds as described in Paget’s seed and soil theory, where metastases develop only when the seed and the soil were compatible. In fact, we envision that DTCs may be active players in modelling the composition of the soil to control their fate. In fact, dormant cells can be found in different organs (bone morrow, lung, liver, brain) suggesting that the soil, different in all of them, cannot be the main player in sustaining their dormancy, and instead, that the “seed” is a major player in the process. We believe that a dynamic reciprocity between the seed and the soil exists, where one influences the other to favor DTC’s fate towards dormancy or proliferation (Figure 1).

## 5. Outstanding Questions and Discussion

**Figure 2:**
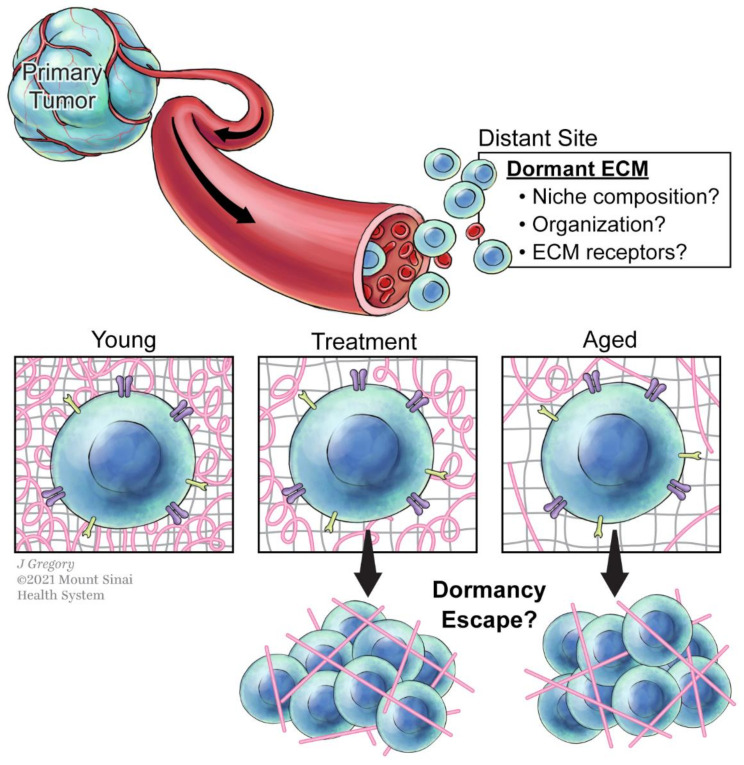
ECM effects on cancer cells during tumor dormancy. Cancer cells (represented in blue) leave the primary tumor and colonize a secondary distant site. In this new site, they encounter new environments that will vary in ECM composition and organization. The repertoire of receptors in these cells may determine their fate, dormancy, or proliferation. Cancer treatments and aging are factors that can influence the ECM architecture of a tissue and influence the escape from dormancy. Collagens are represented in pink, proteoglycans in grey, and ECM receptors in purple and yellow.

Here, we highlight and discuss the open questions in the field that in our view would benefit from a deep analysis in order to increase our knowledge of ECM and tumor dormancy (Figure 2).

### 5.1. What Receptors Are Critical to Sustain Dormancy?

The ECM signals to cells through transmembrane receptors such as integrins, discoidin domain receptors, syndecan, and laminin. Tumor cells that successfully complete the dissemination process and colonize distant organs encounter a new microenvironment with an ECM composition and organization far different from the primary tumor. Their ability to survive as DTCs will depend on the repertoire of receptors they will express to establish adhesion to the new ECM components and trigger signals to engage a dormancy program. We need to understand the contribution of these different receptors to the dormancy state in order to identify targeted therapies that will break the bonds between DTCs and ECM and will induce programmed suicide of these DTCs by releasing them from their niches.

### 5.2. What Is the Composition of a Dormancy ECM Niche?

Another area that lacks information is the ECM composition and architecture in the metastatic site. The ability to perform quantitative mass spectrometry on small samples could be a way to more deeply understand ECM composition of lungs, brain, liver, and other metastatic niches that represent either proliferative or dormant states.

### 5.3. How Does the Matrisome Change with Cancer Treatments?

Treatments of cancer patients include radiation, a plethora of chemotherapy agents, and new treatments using immunotherapies. We need to investigate the impact of these treatments on ECM composition, organization, and stiffness in order to highlight potential side effects that could benefit DTC survival or reactivation in the near or far future of patient lives. For instance, it has been demonstrated that treatment with Tamoxifen can decrease fibronectin and increase collagen I levels in rat mammary glands, thereby providing a tumor suppressor microenvironment that reduces cancer progression [77].

Recently, Su Bin Lim et al. showed that matrisome abnormalities can be predictive of responses to immunotherapy [78], suggesting there may be a link between immunotherapies and the matrisome. Moreover, recent work proposed that the ECM can be modified to increase the efficiency of existing therapies. ECM targeted chemotherapies use the ECM as a target to deliver chemotherapies [79].

### 5.4. How Does Aging Affect the Matrisome and the Scape from Dormancy?

Finally, what so far seems to be the biggest challenge is to understand how ECM changes over time influence DTC survival and awakening to promote metastasis outgrowth. Between primary tumor treatment and metastasis outbreak, several decades can pass in a patient’s life. Recent work by Weeraratna’s laboratory showed that an aged microenvironment can induce metastasis in melanoma models. In their studies, they showed that a decrease in HAPLN1 and the remodeling of collagen in the skin during aging stimulates melanoma metastasis [80,81]. It is well known that ECM composition is affected by aging [82,83] and it has previously been proposed that stimulation of collagen production may rejuvenate ECM and slow aging. This strategy may also have merit for restraining progression to metastasis [84].

### 5.5. Will Remodeling the ECM Microenvironment Prevent Metastasis?

The development of collagen patches to treat wounds has been shown to improve healing, highlighting the potential of bio-printed and engineered ECM scaffolds to treat disease. In an analogy, we can envision a new way to reduce local relapse of cancers by modifying the ECM microenvironment [85,86,87]. Enriching ECM components that promote dormancy will favor an anti-proliferative microenvironment that would prevent tumor cell awakening. ECM remodeling to induce a growth suppressive microenvironment is an exciting idea that could potentially prevent metastatic growth. By identifying ECM components responsible for DTC dormancy or awakening it would be possible to design drugs designed to modify the ECM and prevent DTC proliferation.

Numerous drugs targeting the ECM have been described in the context of cancer (Huang et al. [88] is an extensive review on the topic of therapeutic approaches to target ECM in cancer). One could envision that agents targeting the ECM of dormant cells could prevent their awakening or eliminate them, however a detailed description of the ECM matrisome of these dormant cells is needed to identify potential candidates that could be used to eliminate dormant cells.

## 6. Conclusions

We have reviewed some of the latest concepts in the field of ECM and their relevance to dormancy. We would like to finish our perspective article with two main messages: (1) A further understanding of ECM composition during different stages of cancer metastasis, and in particular the dormancy stage, as well as how ECM changes after cancer treatment, will further expand our understanding of how the ECM is modified during cancer disease and will help us to devise ways to prevent metastatic progression by targeting the ECM. (2) A profound understanding of the source of ECM components (tumor cells, macrophages, fibroblasts) will yield new and more specific targets that could potentially be used for future therapies that target the components of the ECM.
